# Evaluating the rehabilitative and assistive impact of the Agilik exoskeleton: a pre–post single-group study in children with cerebral palsy

**DOI:** 10.3389/fmed.2026.1814298

**Published:** 2026-09-09

**Authors:** Roberta Nossa, Giorgia Malerba, Anna Falivene, Eleonora Diella, Riccardo Riboni, Valentina Grasso, Luca Emanuele Molteni, Mattia Chiappini, Fabio Alexander Storm, Cristina Maghini, Emilia Biffi

**Affiliations:** 1 Scientific Institute, IRCCS Eugenio Medea, Bosisio Parini, Lecco, Italy; 2 Department of Electronics Information and Bioengineering, Politecnico di Milano, Milan, Italy; 3 Department of Psychology, Univeristà degli Studi di Torino, Turin, Italy

**Keywords:** adjustable exoskeleton, cerebral palsy, children rehabilitation, crouch gait, exoskeleton, motorized exoskeleton, wearable exoskeleton, assistive technology

## Abstract

**Introduction:**

Cerebral palsy (CP) is a common childhood-onset neurological condition often associated with crouch gait, a pathological walking pattern impairing mobility. Wearable robotic devices are emerging as promising tools for paediatric gait rehabilitation, but evidence on their rehabilitative and assistive effects remains limited. This exploratory study evaluated the rehabilitative and assistive effects of a 10-session training programme using Agilik, a paediatric knee exoskeleton, in children with CP and crouch gait, during level-ground, uphill, and downhill walking.

**Methods:**

Eight children with CP completed the intervention. Assessments were performed at baseline (T0) and post-intervention (T1), including walking endurance, speed, balance, spasticity, and clinical joint and muscle length assessments. Three-dimensional gait analysis (GRAIL system) quantified sagittal-plane hip, knee, and ankle range of motion during level-ground, uphill, and downhill walking. At T1, joint range of motion was analysed with and without Agilik to assess its assistive effect. User satisfaction was evaluated using the child-adapted QUEST 2.1 questionnaire.

**Results:**

Given the exploratory nature of this study, results were interpreted primarily at the individual level using predefined minimal clinically important difference (MCID) thresholds. When clinically relevant changes occurred, improvements were more frequently observed than deteriorations in walking endurance (MCID > 15 m), speed (MCID > 0.1 m/s), muscle lengths (>5°), and joint range of motion (>5°). No significant group-level rehabilitative changes were observed (all *p* > 0.05). In contrast, the assistive evaluation showed statistically significant increases in knee and hip range of motion across all walking conditions (*p* < 0.05), with all participants improving at the knee in every condition. Satisfaction scores (7-point scale) were high for size, appearance, usability, and set-up time (median ≥ 4/7), while weight, reliability, and timely performance received lower ratings (median 3.5/7).

**Conclusions:**

This exploratory study demonstrates a clear assistive biomechanical benefit of Agilik, particularly at the knee and hip. Individual-level analyses suggest that some participants may experience clinically meaningful improvements following training; however, these observations are hypothesis-generating and cannot be interpreted as evidence of a rehabilitative effect. Larger randomised controlled studies are needed to determine whether repeated exposure to assisted gait translates into sustained functional gains.

## 1 Introduction

Cerebral palsy (CP) is a non-progressive neurodevelopmental condition and represents the leading cause of motor disability in childhood (1, 2). Limitations in functional mobility are common and are typically classified using the Gross Motor Function Classification System (GMFCS). Among ambulatory children with CP, spastic diplegia is the most prevalent subtype and is frequently associated with crouch gait, a pathological gait pattern primarily characterized by excessive knee flexion during the stance phase and often accompanied by excessive hip flexion, insufficient ankle plantarflexion, and other lower-limb deviations such as hip adduction or internal rotation (3–5). Beyond its kinematic definition, crouch gait represents the clinical manifestation of a complex interaction between primary neurological impairments—including spasticity and impaired selective motor control—and secondary musculoskeletal factors, such as muscle weakness, muscle contractures, bone deformities, altered lever arms, and, in some individuals, previous orthopedic interventions (6–9). These interacting factors contribute to reduced walking efficiency, increased energy expenditure, and progressive functional decline (10, 11).

Although CP is non-progressive, gait function often deteriorates over time due to the interaction of primary neurological impairments, such as muscle weakness, spasticity, and impaired motor control, and secondary musculoskeletal changes. Extensor muscle weakness, particularly at the hip, knee, and ankle, is widely recognized as one of the major contributors to the development and progression of crouch gait, especially as body mass increases with growth (12). Conventional non-surgical interventions, including physical therapy, orthoses, and pharmacological treatments, are commonly adopted, while orthopedic surgery is reserved for selected cases (11).

In recent decades, a wide range of robotic devices has been developed for gait rehabilitation in paediatric patients with neurological disorders, including CP (13). Early approaches primarily relied on body-weight-supported treadmill (BWST) systems, which aim to promote repetitive and task-specific stepping practice (14). Advanced robotic platforms integrating BWST, such as Lokomat, Walkbot and Robogait, provide a highly controlled and safe environment for gait training and have been extensively investigated in clinical and research settings (15, 16). Although these systems have demonstrated potential benefits in terms of gait kinematics and functional outcomes, their effectiveness compared to conventional therapy remains debated, and their use is largely limited to specialized clinical settings (15, 16).

These limitations have driven the development of more lightweight and portable robotic solutions, designed to extend gait assistance beyond the clinic and into everyday and home settings (17). Such devices were initially conceived primarily as assistive technologies to support walking in real-life contexts; however, their capacity to deliver task-specific, repetitive, and adaptive support also makes them promising tools for rehabilitation, particularly in non-clinical environments.

These systems can be broadly classified into mobile robotic devices that move with the user, and fully wearable exoskeletons designed to provide joint-specific assistance. Mobile frame-based systems, such as CP-Walker, NF-Walker, Explorer MB and ATLAS 2030, combine robotic actuation with a supporting frame that travels alongside the child, offering substantial postural support and stability during walking (18–20). While these systems may be beneficial for children with more severe motor impairments, their size, weight, and mechanical complexity make them relatively cumbersome and less suitable for use outside supervised clinical environments. Fully wearable exoskeletons represent a more streamlined alternative and can be further differentiated based on the anatomical level of assistance. Pelvic-based devices (e.g., HAL, Angel Legs) provide multi-joint support and enhanced trunk stability, but are generally heavier and more intrusive, which may limit comfort, acceptance, and feasibility for prolonged or home-based use (21–26). Ankle-focused devices (e.g., ReLink-ANK) target distal joint function and may improve push-off mechanics (27–29); however, they do not directly address knee flexion–extension deficits, which are central to the pathophysiology of crouch gait. Knee exoskeletons offer a targeted and potentially more efficient solution for children with crouch gait by directly assisting knee extension during gait phases. Examples include Myosuit, WAKE-up, P.REX, and its subsequent evolution, Agilik (30–38). These devices are generally lighter and less restrictive, allowing more natural overground walking. From both a functional and acceptability perspective, slimmer and less obtrusive devices are more likely to be well tolerated by paediatric users, particularly when assistive support is required during daily activities or extended rehabilitation programs. This consideration is especially relevant when envisioning rehabilitation pathways that extend beyond the clinic into home and community settings.

To date, most studies investigating knee exoskeletons in children with CP have primarily focused on their assistive effects, reporting improvements in gait kinematics, knee extension, and walking efficiency during device use (30–38). Evidence regarding their rehabilitative impact, defined as sustained improvements observed after training and assessed without the device, remains limited. A recent protocol by Devine et al. proposed a home-based rehabilitative intervention using the wearable knee exoskeleton Agilik (39); however, results on the long-term rehabilitative effects of powered, wearable knee exoskeletons in paediatric populations are still lacking.

In this context, there is a clear need for clinical studies specifically designed to investigate not only the assistive but also the rehabilitative potential of wearable knee exoskeletons, which represents the main rationale of the present study. The aim of this study was therefore to assess the rehabilitative effects of Agilik, a CE-marked and FDA-approved wearable knee exoskeleton designed to provide individualized assistance or resistance during gait, based on each child's specific motor impairments and therapeutic goals. This evaluation was conducted during a structured clinical training program in children with CP and crouch gait, using a pre–post single-group study design. Given the novelty of powered paediatric knee exoskeletons and the limited available evidence on their rehabilitative effects, this study was conceived as a exploratory investigation to generate preliminary data and inform the design of future controlled trials. Additionally, beyond investigating the rehabilitative impact, the study also aimed to evaluate the assistive effect of Agilik, thereby extending findings from previous studies that used this device. In particular, this investigation assessed gait including uphill and downhill walking, rather than focusing solely on overground walking.

## 2 Material and methods

### 2.1 Study design and general protocol

This exploratory study was designed as a pre–post single-group clinical study to investigate the rehabilitative effects of a structured intervention using the paediatric lower-limb exoskeleton Agilik in children with CP, as well as to explore the assistive contribution of the device at the end of the training programme. Participants were enrolled according to predefined inclusion and exclusion criteria, detailed in Sec. 2.2.

The study protocol comprised several sequential phases: enrolment, baseline assessments, exoskeleton fitting and configuration, a structured training period of four to five weeks, and post-intervention outcome assessments. During the enrolment phase, potential participants were screened for eligibility. Once inclusion criteria were met, demographic and clinical data were collected, including age, sex, weight, diagnosis, and GMFCS level.

Baseline assessments (T0) were conducted during a dedicated visit lasting up to six hours and included a comprehensive physical examination and instrumental and clinical evaluations, as detailed in Sec. 2.4 (Endpoints and variables to be measured).

The intervention phase consisted of three visits at the orthopedic center dedicated to exoskeleton preparation, including casting for brace fabrication, system setup, and calibration. This was followed by ten training sessions with Agilik, delivered over approximately four to five weeks (two sessions per week). Each session lasted up to one hour and involved walking practice with the exoskeleton in both indoor and outdoor setting, including uphill and downhill walking and stairs climbing. The number of sessions was determined by two complementary considerations: (i) clinical and logistical practicability for an outpatient cohort, several of whom travelled from distant locations, requiring a training volume that was both clinically informative and acceptable in terms of family commitment; and (ii) alignment with the in-lab training protocol described by Devine et al. (39), which adopted 10 visits as the reference standard for in-clinic exoskeleton training with the same device.

Device input parameters were first configured following the manufacturer's recommendations, then progressively adjusted to the individual gait pattern (see Sec. 2.3 Equipment for more details) and the therapists' clinical judgment.

Throughout the intervention period, participants were instructed to continue their usual standard of care, including their ongoing rehabilitative and/or sport programmes and routine clinical management. No planned modifications specifically targeting cerebral palsy (e.g., additional physiotherapy programmes, changes in orthotic management, antispastic pharmacological treatments) were introduced during the intervention. No botulinum toxin injections and/or orthopedic lower limb surgery were reported in the previous year. Therefore, the Agilik training represented the only planned modification to participants' treatment throughout the intervention.

Post-intervention assessments (T1) were conducted using the same protocol adopted at baseline. In addition to the evaluation performed without the exoskeleton, an assistive assessment was carried out at T1 by repeating selected outcome measures with Agilik worn and operating in assistive mode, in order to quantify the margin of improvement attributable to the device.

The study was approved by the Ethics Committee of IRCCS E. Medea—Sezione Scientifica Associazione La Nostra Famiglia (Prot. N. 75/22—CE, 28/12/2022). Written informed consent was obtained from all legal guardians prior to enrolment, and participants were expected to comply with study procedures for its entire duration.

### 2.2 Participants

Children were enrolled at IRCCS E. Medea (Bosisio Parini, Italy) to participate in this study. Eligibility was determined based on a set of predefined inclusion and exclusion criteria.

Inclusion criteria were children with a confirmed diagnosis of CP, specifically those presenting spastic diplegia and exhibiting a crouch gait. Eligible participants were aged 5 to 17 years, weighed less than 70 kg, and had a knee flexion contracture of less than 10° in the supine position. Children were also required to have no hamstring contracture, as assessed by the straight-leg-raising test, and no tibio-tarsal arthrodesis. Participants needed to be able to walk at least 3 meters without stopping, with or without a walking aid, and to understand and follow simple instructions, as verified by parent report and clinician observation. Functional ability was restricted to GMFCS levels I–III, and Modified Ashworth Scale (MAS) scores ≤2 were required for each lower-limb muscle group.

Exclusion criteria included severe neurological, musculoskeletal, or cardiorespiratory conditions that would impair walking, history of uncontrolled seizures within the past year, severe spasticity, GMFCS level IV or V, and hip or knee flexion contractures exceeding 10°.

### 2.3 Equipment

Agilik is a motorized orthotic system specifically designed to support or resist knee movements throughout the different phases of gait. The device is usually applied bilaterally, with one orthosis per leg, and is capable of delivering up to 12 Nm of torque at the knee joint, in either flexion or extension.

The system consists of custom-molded Knee-Ankle-Foot Orthoses (KAFOs), tailored to each patient, equipped with integrated electro-mechanical actuators, a battery pack, cabling, and dedicated software (Agilik App). Embedded foot pressure sensors within the KAFO footbed, together with measurements of knee angular velocity, allow the device to detect gait phases in real time, including early, mid and late stance, as well as early and late swing. This enables the controller to provide phase-specific torque: extension torque is typically delivered during initial and mid-stance as well as late swing, whereas flexion torque is delivered during initial swing. Depending on the individual gait pattern, additional flexion torque may be delivered during late stance.

Personalization of assistance is achieved through the Agilik App, a Bluetooth-connected software interface that enables clinicians and biomedical engineers to adjust control parameters in real time. Adjustable parameters include:

- Foot-pressure voltage thresholds (mV), specifically the Initial Contact (IC) and Toe-Off (TO) thresholds, which define the sensor signal levels required to detect foot contact (stance phase) and foot release (swing phase), respectively.
- Knee angular velocity thresholds (deg/s), which regulate transitions between gait sub-phases (early–mid stance, mid–late stance, and early–late swing). Early stance corresponds to knee flexion during loading response; the transition to mid-stance is triggered when angular velocity shifts from flexion to extension, enabling forward progression. The mid-to-late stance transition occurs when the knee moves from extension into pre-swing flexion. During swing, the early-to-late transition is identified when knee motion shifts from flexion to extension.
- Torque ramp rates (Nm/s), which determine how rapidly torque increases toward (ramp-on) or decreases from (ramp-off) the preset phase-specific target value. Lower ramp rates produce smoother and less perceptible torque modulation, whereas higher ramp rates result in faster, more pronounced transitions and generally greater effective support.
- Phase-specific torque amplitudes (Nm), independently configurable for flexion and extension within a ± 12 Nm range (negative values = extension; positive values = flexion).

These parameters were progressively fine-tuned across sessions according to each child's gait pattern, knee strength, comfort, and therapeutic goals, allowing optimisation of assistance while minimising the risk of overexertion. Initial values were set according to the manufacturer's recommended defaults as specified in the device manual, and subsequently individualised for each participant across sessions. Adjustments were guided by clinical observation of the child's gait pattern during device use, in a structured dialogue between the supervising biomedical engineer, responsible for operating the Agilik App, and the treating clinician, who evaluated gait quality and identified specific targets for improvement. Software-defined knee angle limits further constrained the joint range within which torque could be delivered, providing an additional safety safeguard against excessive extension.

Agilik is classified as a Class I medical device suitable for both clinical and home use, and its operation complies with IEC 60601-1 standards for non-continuous use.

### 2.4 Endpoints and variables to be measured

At baseline (T0), demographic and clinical characteristics were recorded, including age, sex, weight, and functional level according to the GMFCS (40).

Given the exploratory nature of the study, no primary or secondary outcomes were pre-specified. A comprehensive set of clinical and instrumental measures was collected at baseline (T0) and after the intervention period (T1) to explore both rehabilitative and assistive effects. The outcome measures included:

- Gait endurance measured by the Six-Minute Walk Test (6MWT), which measures the maximum distance a participant can walk over six minutes along a 25 m corridor (41).
- Gait speed, assessed using the Ten-Meter Walk Test (10MWT) (42). Participants performed the test under two conditions: at a comfortable, self-selected speed (usual speed) and at their maximum safe speed (fast speed). Each condition was performed twice, and for the fast-speed condition, the highest value of the two trials was recorded, while for the usual-speed condition, the average of the two trials was used.
- Spasticity of lower-limb muscles graded using the MAS, which quantifies resistance to passive movement on a 0–4 scale, with higher scores indicating increased muscle tone (43). The following muscle groups were evaluated bilaterally: hip flexors, hip adductors, knee flexors, knee extensors, and ankle plantar flexors. Specifically, MAS scores were collected separately for the left and right sides for each muscle group. For data analysis, left- and right-side MAS scores were subsequently averaged to obtain a mean spasticity score for each muscle group.
- Muscle lengths and joint range of motion (ROM) of the lower limbs, measured via physical examination and manual goniometry (44). Muscle length was evaluated for major lower-limb muscles affecting gait, including iliopsoas, rectus femoris, ischiocrural and gastrocnemius muscles. Joint ROM evaluation included knee and ankle movements, specifically focusing on knee extension/flexion and ankle plantarflexion/dorsiflexion.
- Balance and postural control, evaluated through Center of Pressure (COP) displacement recorded using a force platform during static standing (45). Data were collected under four conditions: eyes open with feet apart, eyes open with feet together, eyes closed with feet apart, and eyes closed with feet together. Each condition was repeated three times for 60 s; the most representative trial was then selected by an expert physiotherapist and considered for further analysis. Data were filtered with a fourth-order low-pass Butterworth filter with a cutoff frequency of 15 Hz (46). Sway path (total COP trajectory length) and sway area were then calculated for each condition using a custom-developed MATLAB script.
- Three-dimensional (3D) gait analysis conducted using the GRAIL system (Motek Medical, The Netherlands) during level-ground, uphill (+10° slope), and downhill (−5° slope) walking. Gait analysis was performed using a computerized 3D motion capture system with 10 infrared cameras (Vicon Motion Systems, Oxford, UK) at a sampling rate of 100 Hz. For each participant, 26 reflective markers were attached to the lower limbs according to the Human Body Model (47). For each condition (level-ground, uphill, downhill), participants completed 1 min of familiarization walking, after which 20 steps were recorded. Full-body kinematic data were collected to characterise lower-limb movement patterns across the different walking conditions. Offline processing was performed with the Gait Offline Analysis Tool (Motek, Amsterdam, The Netherlands), which allowed manual deletion of missteps (e.g., gait cycles with foot placement on only one force platform or with non-visible passive markers). Sagittal-plane joint kinematics of the hip, knee, and ankle were extracted for both lower limbs. For each joint and walking condition, joint ROM was calculated over the gait cycle using a custom MATLAB script specifically developed for this study. ROM values were computed separately for the right and left lower limbs and subsequently averaged to obtain a single representative value per participant, joint, and condition. These averaged ROM values were then used for within-subject comparisons between baseline (T0) and post-intervention (T1), as well as for the assistive evaluation performed at T1.

At T0, all assessments were performed without the exoskeleton. At T1, the 3D gait analysis performed with the GRAIL system was collected both without and with Agilik, enabling the distinction between training-related (rehabilitative) effects and the assistive contribution of the device. For gait-related assessments without Agilik, participants wore their usual orthoses if they were accustomed to using them.

Finally, user satisfaction at T1 was evaluated using a child-adapted version of the Quebec User Evaluation of Satisfaction for children (QUEST 2.1: children's version) (48). The questionnaire included 10 items, eight focused on the device and two on the provided service, rated on a seven-point Likert scale from 1 (“not satisfied at all”) to 7 (“very satisfied”). The questionnaire items were as follows:

- Size: satisfaction with the overall dimensions of the device and its fit for the user.
- Weight: satisfaction with the device's weight and perceived heaviness during use.
- Easy to move: ease of moving or walking while wearing the device.
- Appearance: aesthetic appeal and visual acceptability of the device.
- Easy to use: overall usability and simplicity of operating the device.
- Set-up time: satisfaction with the time required to configure and prepare the device for use.
- Reliability: perception of the device's consistent performance during use.
- Timely performance: responsiveness and promptness of the device during operation.
- Support satisfaction: satisfaction with the support provided by the clinical or technical staff.
- Training satisfaction**:** satisfaction with the training received for using the device.

To obtain domain-specific scores, items related to the device (*n* = 8) and service (*n* = 2) were first aggregated at the individual level by computing the median score per participant, and then summarized at the group level using the median across participants.

Study data were collected and managed using REDCap (Research Electronic Data Capture), a secure, web-based platform specifically developed to support research data acquisition and management. The system provides structured and validated data entry interfaces, ensures traceability of data through audit logs, and allows efficient export of datasets to standard statistical software packages (49, 50).

### 2.5 Statistical analyses

All statistical analyses were performed using SPSS software (IBM SPSS Statistics 21.0). Descriptive statistics were used to summarise demographic characteristics and dropout rates. Data normality was assessed using the Shapiro–Wilk test. Data are presented as mean ± standard deviation (SD) for parametric variables and as median (interquartile range, IQR) for non-parametric variables. Statistical significance was set at *p* < 0.05.

Changes in clinical and instrumental outcomes from baseline (T0) to post-intervention (T1) were evaluated using within-group comparisons. For the assistive evaluation, comparisons were conducted at T1 between conditions without and with the exoskeleton to quantify the assistive effect of Agilik. Paired-sample *t*-tests were performed for normally distributed variables, whereas the Wilcoxon signed-rank test was used for variables that did not meet normality assumptions.

Differences among questionnaire items were assessed using the Friedman test for related samples. When statistically significant, *post hoc* pairwise comparisons were conducted using Wilcoxon signed-rank tests with Bonferroni–Holm correction for multiple comparisons.

Given the exploratory nature of the study, no formal sample size calculation based on a predefined primary outcome was performed. The aim was to evaluate the practicality and preliminary rehabilitative effects of training with Agilik in a clinical paediatric population, as well as to inform the design of future controlled trials.

## 3 Results

Between 2 March 2023 and 21 May 2025, a total of nine children with CP and crouch gait were enrolled at IRCCS E. Medea to participate in this study (4 females, 5 males). One participant (female, GMFCS level II, 9.4 years old) withdrew from the study before completing the intervention and was therefore considered a dropout. Participants' demographic and clinical characteristics, together with information on ongoing physiotherapy, stretching programmes, and orthotic management, are summarised in Table 1. The median age of the participants was 11.85 (4.73) years, and the median body weight was 39 (11.38) kg. According to the GMFCS, two participants were classified as level I, four as level II, and two as level III. No participant received oral antispastic medication or botulinum toxin injections during the study period. Participant 6 was receiving daily carbamazepine as an ongoing antiepileptic treatment, which remained unchanged throughout the study.

**Table 1:** Demographics and clinical characteristics.

P	Sex	Age (years)	Weight (kg)	GMFCS level	Ongoing physiotherapy	Stretching programme	Orthotic management
1	M	10.5	42	III	PT + aquatic therapy + NPS support (1 week)	Yes	Bilateral AFOs + 1 walking aid
2	F	8.3	23	II	PT (2–3 sessions/week)	Yes	Bilateral AFOs
3	M	16.5	45	I	Sport (2 sessions/week)	No	None
4	F	17	45	I	None	No	Bilateral insoles
5	M	15	52	II	PT (6–7 sessions/week)	Yes	Bilateral AFOs
6	M	10.7	35.5	II	None	Standing frame use	Bilateral AFOs
7	F	11	28	II	PT (1 session/week) + sport	Yes	Bilateral Saebo splints + Flexa suit
8	M	12.7	36	III	PT (3 sessions/week)	Yes	Bilateral AFOs + 2 walking aids

P, participant; PT, physiotherapy; NPS, neuropsychological.

Given the exploratory nature of this study, the small and heterogeneous sample, and the absence of a predefined primary outcome, results were reported and interpreted primarily at the individual level, using minimal clinically important difference (MCID) thresholds. MCID thresholds were selected from the published literature and, where available, from studies conducted specifically in children with cerebral palsy. We identified a threshold of 15 m for the 6MWT from a study in children with motor impairment (51), including CP. For the 10MWT, we adopted an MCID of 0.1 m/s (52), whereas a 5° threshold was used for joint range of motion and muscle length (53); both thresholds were derived from studies conducted in children with CP.

Group-level pre–post statistical comparisons are reported alongside as supplementary, descriptive context rather than as the primary basis for inference. For each outcome, individual response patterns (improvement, decline, or stability) are summarised in Tables 2–9 and in the accompanying Supplementary File (case-by-case workbook), which also include individual T0/T1 values for every outcome.

**Table 2:** Individual data for the 6MWT.

**Participant**	**6MWT T0 (m)**	**6MWT T1 (m)**	**Δ (m)**	**6MWT response (MCID = 15 m)**
**1**	339.7	330.4	−9.3	No change
**2**	403.8	412	+8.2	No change
**3**	502.5	540	+37.5	Improved
**4**	429	466	+37.0	Improved
**5**	328	400	+72.0	Improved
**6**	407	367	−40.0	Worsened
**7**	432.2	418	−14.2	No change
**8**	400	394	−6.0	No change

**Table 3:** Individual data for the 10MWT.

**Participant**	**Δ 10MWT (m/s) Std condition**	**Std response (MCID = 0.1 m/s)**	**Δ 10MWT (m/s) Fast condition**	**Fast response (MCID = 0.1 m/s)**
**1**	−0.323	Worsened	−0.339	Worsened
**2**	+0.029	No change	−0.044	No change
**3**	+0.111	Improved	+0.176	Improved
**4**	+0.113	Improved	+0.175	Improved
**5**	+0.196	Improved	+0.092	No change
**6**	−0.304	Worsened	−0.034	No change
**7**	−0.134	Worsened	−0.049	No change
**8**	+0.240	Improved	+0.070	No change

Std, standard.

**Table 4:** Individual data for the MAS.

**Participant**	**Δ MAS left**	**Left response**	**Δ MAS right**	**Right response**
**1**	+0.10	Worsened	+0.00	No change
**2**	−0.40	Improved	−0.60	Improved
**3**	−0.20	Improved	+0.00	No change
**4**	−0.60	Improved	−0.60	Improved
**5**	−0.10	Improved	−0.20	Improved
**6**	+0.10	Worsened	+0.20	Worsened
**7**	+0.20	Worsened	+0.30	Worsened
**8**	+0.10	Worsened	+0.00	No change

**Table 5:** Per-participant trend for the muscle lengths.

**P**	**Iliopsoas L resp**	**Iliopsoas R resp**	**Rectus femoris L resp**	**Rectus femoris R resp**	**Ischio L resp**	**Ischio R resp**	**Gastro L resp**	**Gastro R resp**
**1**	Improved	Improved	Improved	Improved	Improved	Improved	No change	No change
**2**	No change	No change	Improved	Improved	No change	No change	No change	No change
**3**	No change	Improved	Improved	Improved	Improved	Improved	No change	No change
**4**	No change	No change	Worsened	Worsened	Improved	Improved	Improved	Improved
**5**	Improved	Improved	Worsened	No change	Improved	Improved	No change	No change
**6**	No change	No change	No change	No change	Worsened	Worsened	Improved	Improved
**7**	No change	No change	No change	Worsened	No change	No change	Improved	No change
**8**	Improved	Improved	Improved	Improved	Worsened	No change	Improved	No change

P, participant; L resp, left response; R resp, right response; Ischio, Ischiocrural; Gastro, Gastrocnemius.

**Table 6:** Per-participant trend for the joint ROMs.

P	Knee ext ROM L (T0 → T1)	Knee ext L resp.	Knee ext ROM R (T0 → T1)	Knee ext R resp.	Ankle DF ROM L (T0 → T1)	Ankle DF L resp.	Ankle DF ROM R (T0 → T1)	Ankle DF resp.
**1**	0° → 0°	No change	2° → 0°	No change	20° → 7°	Improved	34° → 25°	Worsened
**2**	0° → 0°	No change	2° → 1°	No change	5° → 19°	Improved	2° → 13°	Improved
**3**	−4° → −5°	No change	−5° → 2°	Improved	15° → 14°	No change	5° → 8°	No change
**4**	4° → 2°	No change	8° → 4°	No change	16° → 14°	No change	21° → 15°	Worsened
**5**	−8° → −6°	No change	−8° → −9°	No change	20° → 15°	Worsened	18° → 15°	No change
**6**	−7° → 0°	Improved	−8° → 0°	Improved	6° → 12°	Improved	13° → 12°	No change
**7**	3° → 1°	No change	0° → 0°	No change	22° → 22°	No change	17° → 16°	No change
**8**	−2° → −3°	No change	−1° → −3°	No change	28° → 30°	No change	28° → 30°	No change

P, participant; Knee Ext, knee extension; Ankle DF, ankle dorsiflexion; L resp, left response; R resp, right response.

**Table 7:** Per-participant trend for the balance.

**P**	**EOFA**	**EOFT**	**ECFA**	**ECFT**	**EOFA**	**EOFT**	**ECFA**	**ECFT**
	**Sway path resp.**	**Sway path resp.**	**Sway path resp.**	**Sway path resp.**	**Sway area resp.**	**Sway area resp.**	**Sway area resp.**	**Sway area resp.**
**1**	Improved	Improved	Improved	Improved	Improved	Improved	Improved	Improved
**2**	Improved	Worsened	Worsened	Worsened	Improved	Improved	Worsened	Improved
**3**	Improved	Improved	Improved	Improved	Worsened	Improved	Improved	Improved
**4**	Improved	Improved	Improved	Improved	Worsened	Worsened	Improved	Worsened
**5**	Improved	Improved	Improved	Improved	Worsened	Worsened	Worsened	Improved
**6**	Worsened	Improved	Improved	Improved	Worsened	Worsened	Improved	Improved
**7**	Worsened	Improved	Improved	Improved	Improved	Worsened	Improved	Improved
**8**	Improved	Worsened	Worsened	Worsened	Worsened	Improved	Worsened	Worsened

P, participant; EOFA, eyes open/feet apart; EOFT, eyes open/feet together; ECFA, eyes closed/feet apart; ECFT, eyes closed/feet together; Resp, response.

**Table 8:** Individual-level patterns for the ROM (rehabilitative effect).

**P**	**Flat ankle resp.**	**Flat knee resp.**	**Flat hip resp.**	**Downhill ankle resp.**	**Downhill knee resp.**	**Downhill hip resp.**	**Uphill ankle resp.**	**Uphill knee resp.**	**Uphill hip resp.**
**1**	No change	Improved	Improved	Improved	No change	No change	No change	Improved	Improved
**2**	No change	No change	No change	Improved	No change	Worsened	No change	Worsened	No change
**3**	Worsened	No change	No change	No change	No change	No change	Worsened	No change	Improved
**4**	No change	No change	No change	No change	No change	No change	No change	Worsened	Worsened
**5**	No change	No change	No change	No change	No change	No change	Improved	No change	No change
**6**	No change	No change	No change	Worsened	No change	Improved	No change	Worsened	No change
**7**	Improved	No change	No change	No change	No change	No change	No change	No change	No change
**8**	Improved	No change	No change	N/A	N/A	N/A	Improved	No change	No change

P, participant; resp, response.

**Table 9:** Individual-level patterns for the ROM (assistive effect).

**P**	**Flat ankle resp.**	**Flat knee resp.**	**Flat hip resp.**	**Downhill ankle resp.**	**Downhill knee resp.**	**Downhill hip resp.**	**Uphill ankle resp.**	**Uphill knee resp.**	**Uphill hip resp.**
**1**	Improved	Improved	Improved	Worsened	Improved	Improved	Improved	Improved	Improved
**2**	Improved	Improved	Improved	Worsened	Improved	Improved	Improved	Improved	Improved
**3**	Improved	Improved	Improved	Improved	Improved	Improved	Worsened	Improved	Worsened
**4**	Improved	Improved	Improved	Improved	Improved	Improved	Worsened	Improved	Improved
**5**	Improved	Improved	Improved	Improved	Improved	Improved	Worsened	Improved	Improved
**6**	Improved	Improved	Improved	Improved	Improved	Improved	Improved	Improved	Improved
**7**	Worsened	Improved	Improved	Improved	Improved	Improved	Improved	Improved	Improved
**8**	Worsened	Improved	Improved	Worsened	Improved	Improved	Worsened	Improved	Improved

P, participant; resp, response.

### 3.1 6MWT results

At the individual level, 3/8 participants (37.5%) showed an improvement in 6MWT distance exceeding the MCID threshold of 15 m (51), 1/8 (12.5%) showed a decline beyond this threshold, and 4/8 (50%) remained stable. Participants 3, 4 and 5 improved, while participant 6 declined; Table 2 reports individual values.

**Figure 1:**
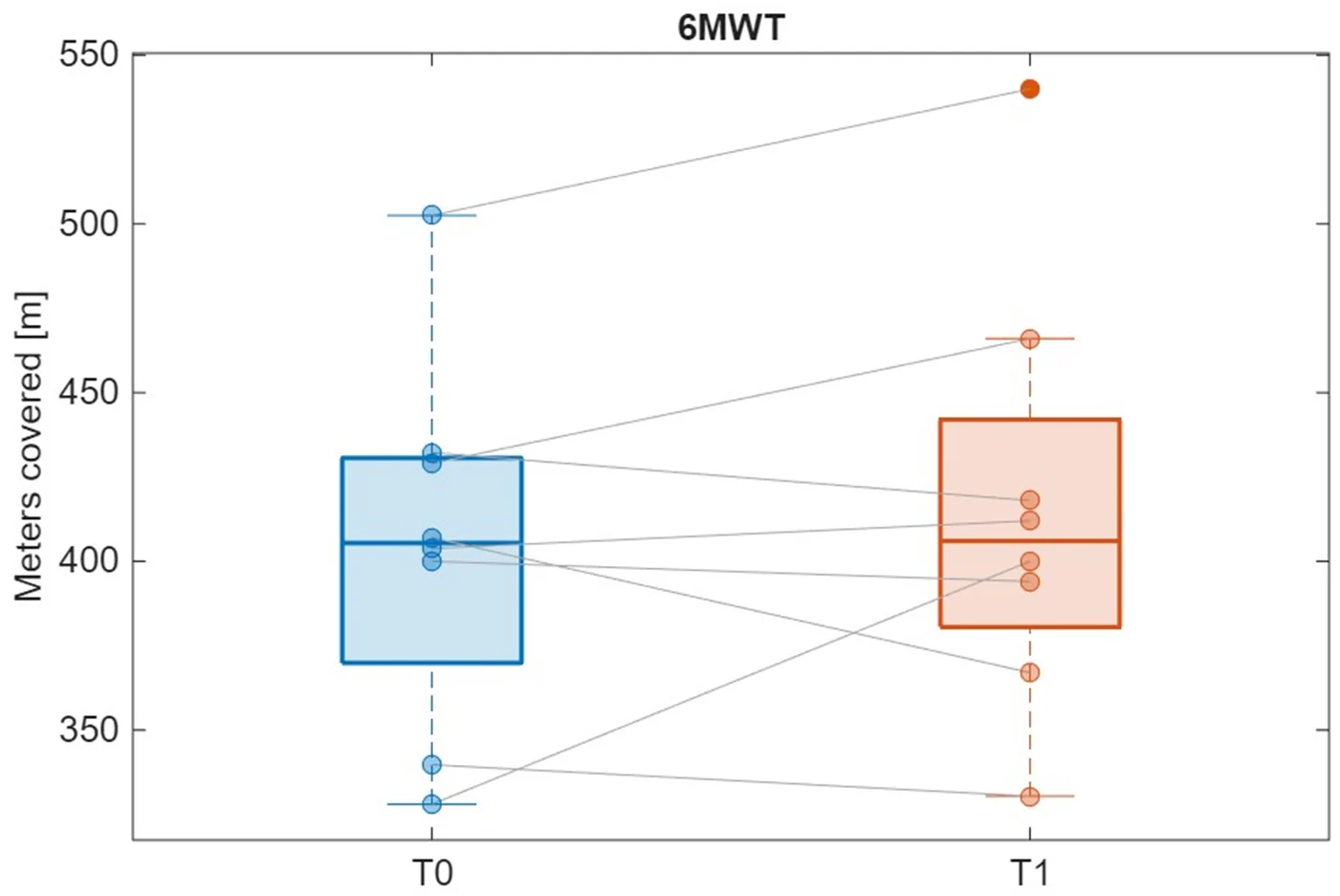
**Boxplot of Six-Minute Walk Test distances at baseline (T0) and post-intervention (T1).** Data at T0 are shown in blue, and data at T1 in orange. Each dot represents an individual participant. Box limits correspond to the 25th and 75th percentiles, whiskers indicate the range of values excluding outliers, and the central line represents the median.

At group level, median gait endurance was 405.40 (44.88) m at baseline (T0) and 406.00 (42.75) m post-intervention (T1); the pre–post comparison did not show a statistically significant difference (*p* = 0.67), consistent with the heterogeneous individual response pattern described above. A boxplot illustrating the distribution of 6MWT distances at T0 and T1, with individual data points, is presented in Figure 1.

### 3.2 10mWT results

10MWT performance was evaluated under both standard and fast walking conditions at baseline (T0) and post-intervention (T1). Using a MCID threshold of 0.1 m/s (52), in the standard walking condition 4/8 participants (50%) showed an improvement exceeding the MCID and 3/8 (37.5%) showed a decline; in the fast walking condition, 2/8 (25%) improved and 1/8 (12.5%) declined according to the same criterion. Individual direction of change by participant is reported in Table 3; notably, participants 3 and 4 improved consistently in both standard and fast conditions, while participant 6, who also declined on the 6MWT, declined in the standard condition as well.

**Figure 2:**
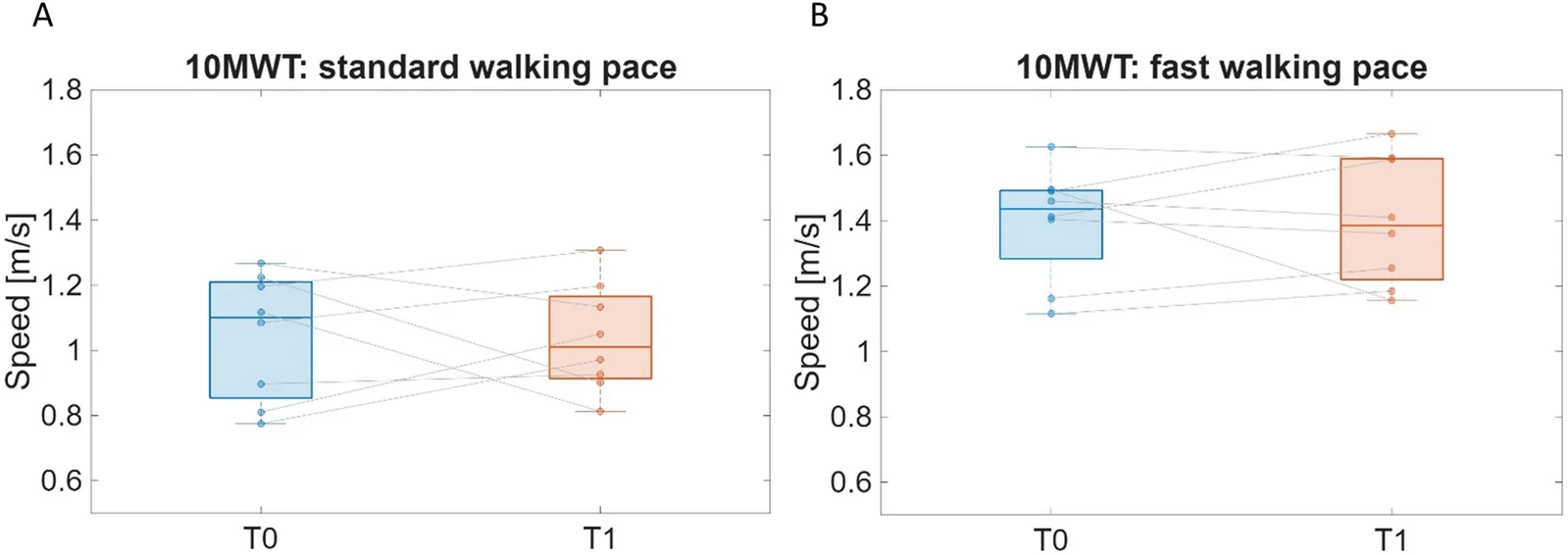
**10MWT performance at baseline (T0) and post-intervention (T1) for standard and fast walking conditions.** Panel (A) shows the boxplot for standard walking, while Panel (B) shows the boxplot for fast walking. In each panel, T0 values are shown in blue and T1 values in orange. Individual subject data are represented by dots overlaid on the boxes. The boxes indicate the interquartile range (IQR), and the horizontal line within each box represents the median gait speed.

At group level, median gait speed in the standard walking condition was 1.10 (0.33) m/s at T0 and 1.01 (0.23) m/s at T1; in the fast walking condition, median gait speed was 1.44 (0.15) m/s at T0 and 1.39 (0.35) m/s at T1. Pre–post comparisons did not show statistically significant differences for either walking condition (*p* > 0.05). A boxplot illustrating individual and group data for both walking conditions is shown in Figure 2.

### 3.3 MAS results

Spasticity was assessed using the MAS, expressed as the average score across muscle groups for each limb. At the individual level, in the left lower limb 4/8 participants (50%) showed a decrease in MAS score and 4/8 (50%) showed an increase; in the right lower limb, 3/8 (37.5%) showed a decrease, 2/8 (25%) showed an increase, and the remaining 3/8 (37.5%) showed no change. Individual direction of change by participant and limb is reported in Table 4; participant 6, who showed clinically relevant decline in the 6MWT and 10mWT, also showed worsening MAS scores bilaterally, consistent with internal measurement coherence for this individual case.

**Figure 3:**
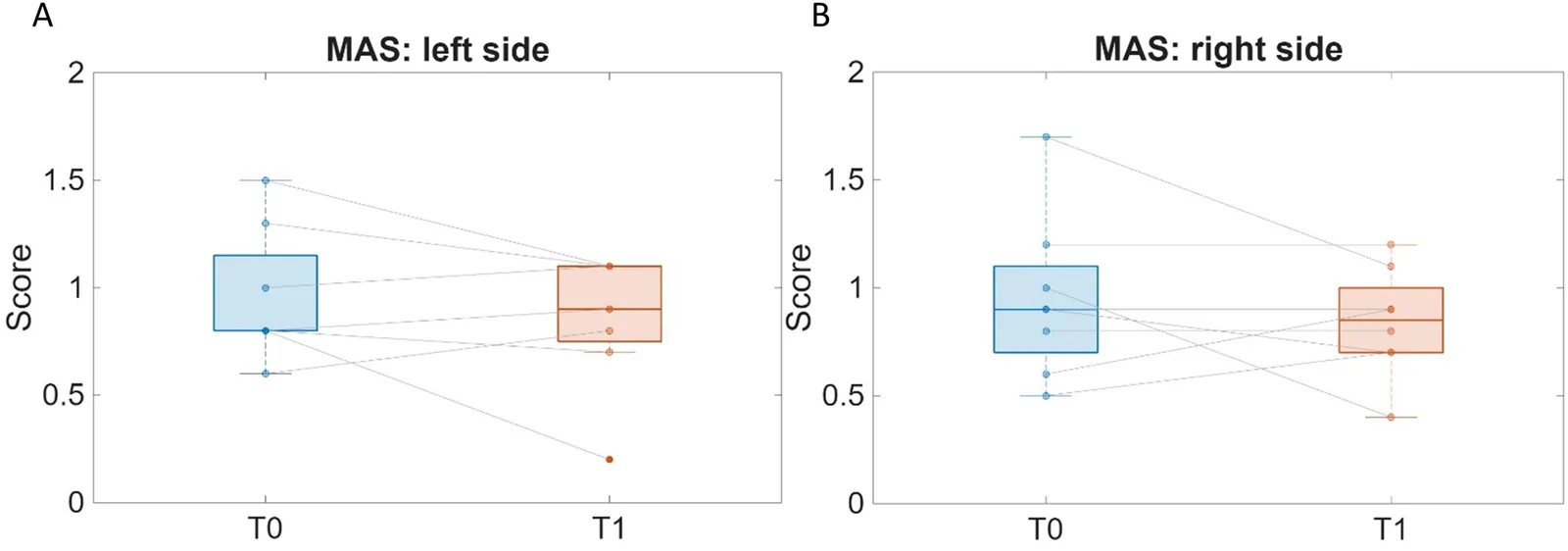
**Boxplots of MAS scores at baseline (T0) and post-intervention (T1).** Panel (A) shows results for the left lower-limb district, while panel (B) refers to the right lower-limb district. Data at T0 are displayed in blue and data at T1 in orange. Each dot represents an individual participant. Boxes indicate the interquartile range (25th – 75th percentiles), the central line represents the median, and whiskers depict the data range excluding outliers.

At group level, no statistically significant changes from T0 to T1 were observed for either the left or right lower-limb district (*p* > 0.05). Median MAS score in the left district was 0.80 (0.28) at T0 and 0.90 (0.33) at T1; in the right district, 0.90 (0.30) at T0 and 0.85 (0.25) at T1. Boxplots illustrating MAS scores at T0 and T1 for both districts are shown in Figure 3.

### 3.4 Muscle length and joint ROM results

#### 3.4.1 Muscle length

Muscle lengths of the iliopsoas, rectus femoris, ischiocrural, and gastrocnemius were assessed bilaterally at baseline (T0) and post-intervention (T1). Individual changes were examined using a 5° threshold as a reference for clinically relevant variation (53); per-participant values and direction of change are reported in Table 5. Response patterns were heterogeneous across muscles and limbs:

- **Iliopsoas**: left district, 3/8 (37.5%) improved, 0/8 worsened; right district, 4/8 (50%) improved, 0/8 worsened.
- **Rectus femoris**: left district, 4/8 (50%) improved, 2/8 (25%) worsened; right district, 4/8 (50%) improved, 2/8 (25%) worsened.
- **Ischiocrural**: left district, 4/8 (50%) improved, 2/8 (25%) worsened; right district, 4/8 (50%) improved, 1/8 (12.5%) worsened.
- **Gastrocnemius**: left district, 4/8 (50%) improved, 0/8 worsened; right district, 2/8 (25%) improved, 0/8 worsened.

Participants 1, 3 and 8 showed broadly coherent bilateral improvement across most muscle groups, while participant 6 showed worsening ischiocrural extensibility bilaterally despite improving on gastrocnemius length and on clinical knee/ankle ROM, indicating that the same participant can show divergent response patterns across different structural and functional domains.

**Figure 4:**
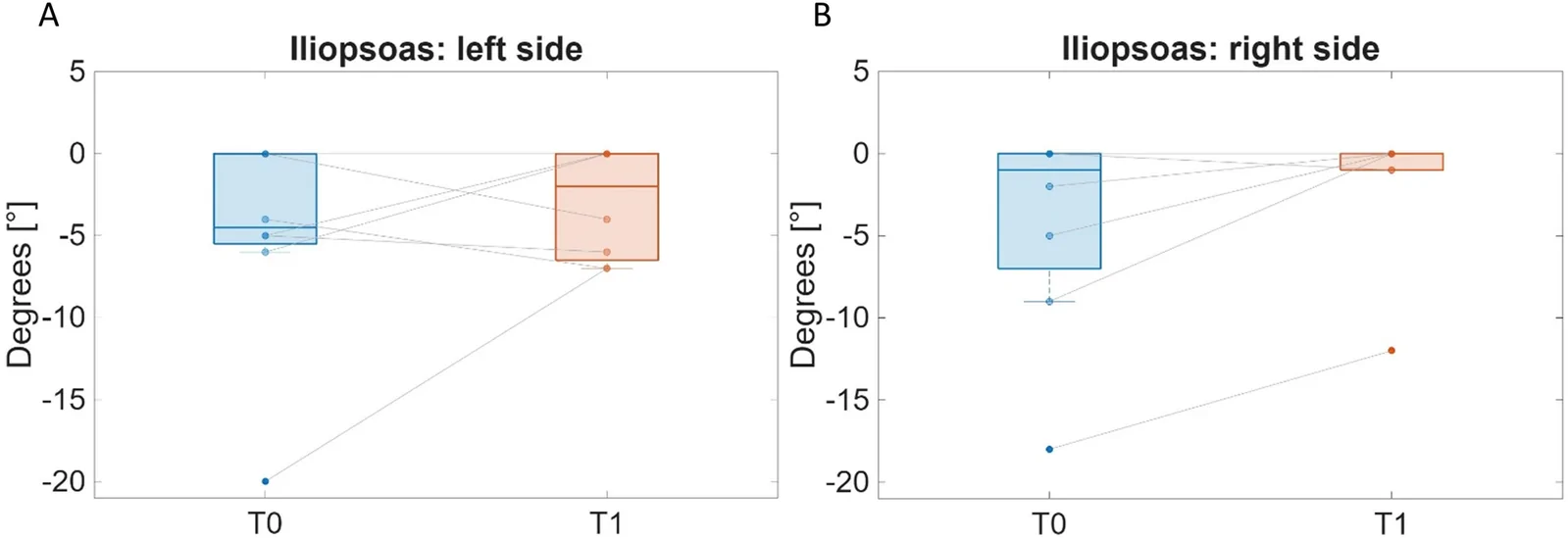
**Boxplots of muscle length measurements at baseline (T0) and post-intervention (T1) for the iliopsoas**. Panel (A) shows results for the left lower-limb district, while panel (B) refers to the right lower-limb district. Data at T0 are displayed in blue and data at T1 in orange. Each dot represents an individual participant. Boxes indicate the interquartile range (25th – 75th percentiles), the central line represents the median, and whiskers depict the data range excluding outliers.

**Figure 5:**
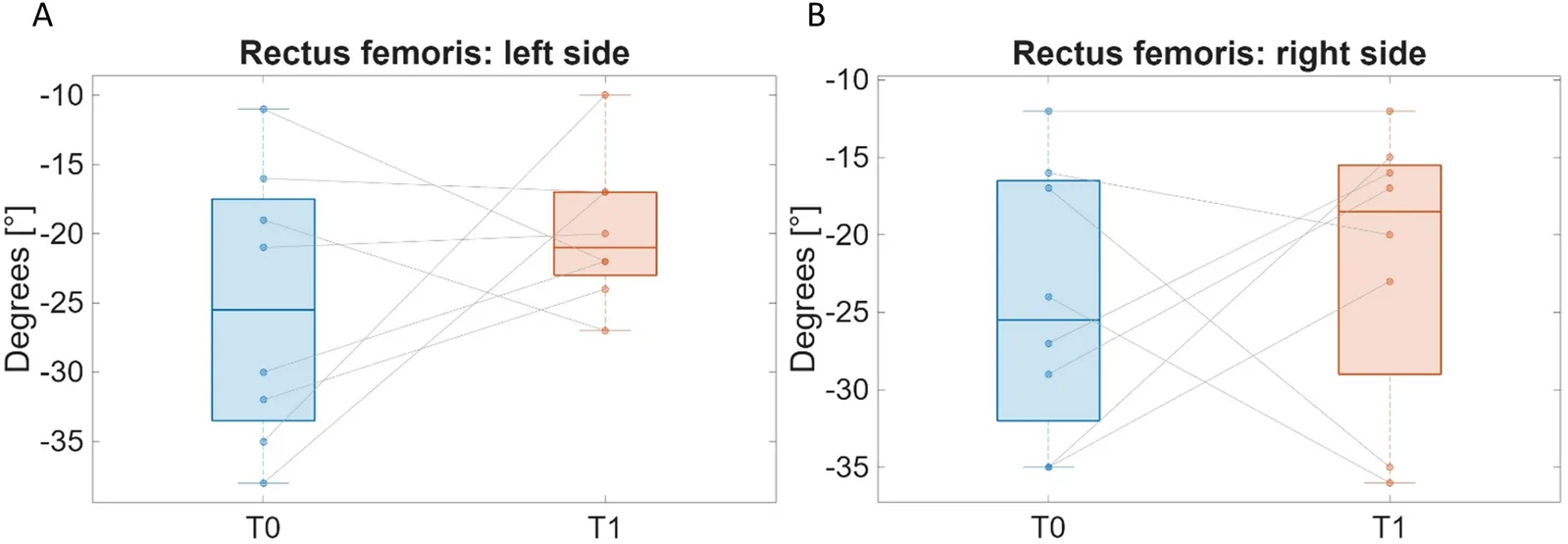
**Boxplots of muscle length measurements at baseline (T0) and post-intervention (T1) for the rectus femoris.** Panel (A) shows results for the left lower-limb district, while panel (B) refers to the right lower-limb district. Data at T0 are displayed in blue and data at T1 in orange. Each dot represents an individual participant. Boxes indicate the interquartile range (25th – 75th percentiles), the central line represents the median, and whiskers depict the data range excluding outliers.

**Figure 6:**
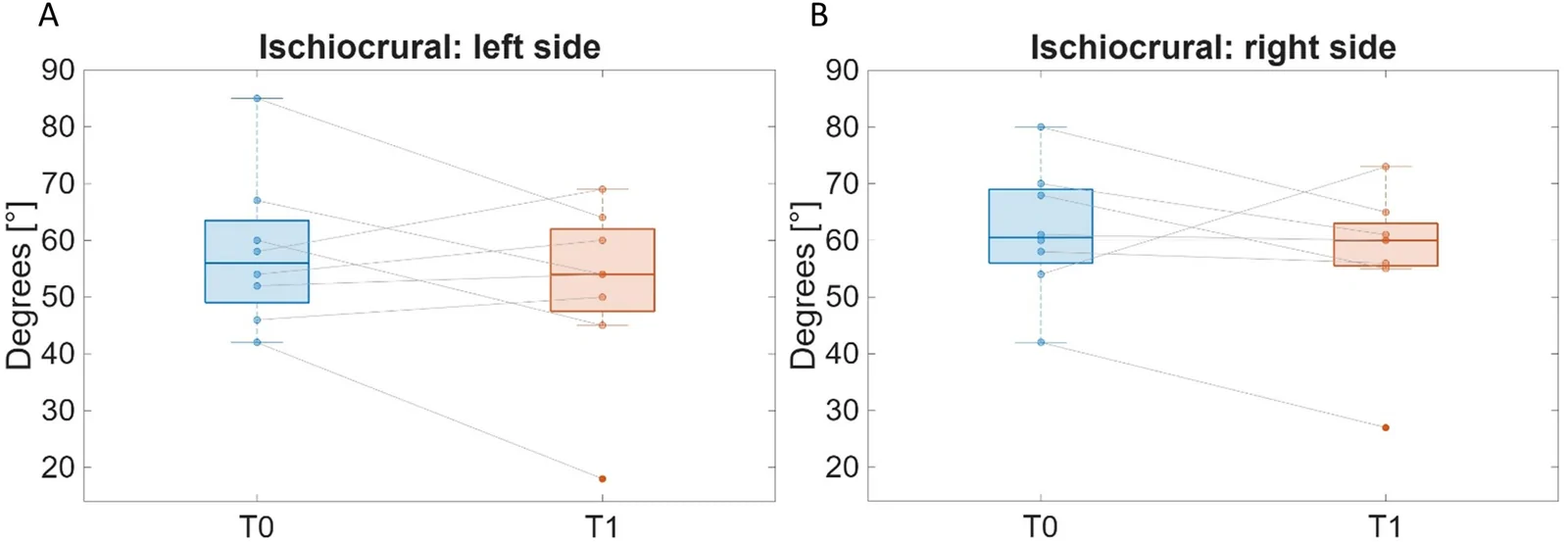
**Boxplots of muscle length measurements at baseline (T0) and post-intervention (T1) for the ischiocrural.** Panel (A) shows results for the left lower-limb district, while panel (B) refers to the right lower-limb district. Data at T0 are displayed in blue and data at T1 in orange. Each dot represents an individual participant. Boxes indicate the interquartile range (25th – 75th percentiles), the central line represents the median, and whiskers depict the data range excluding outliers.

**Figure 7:**
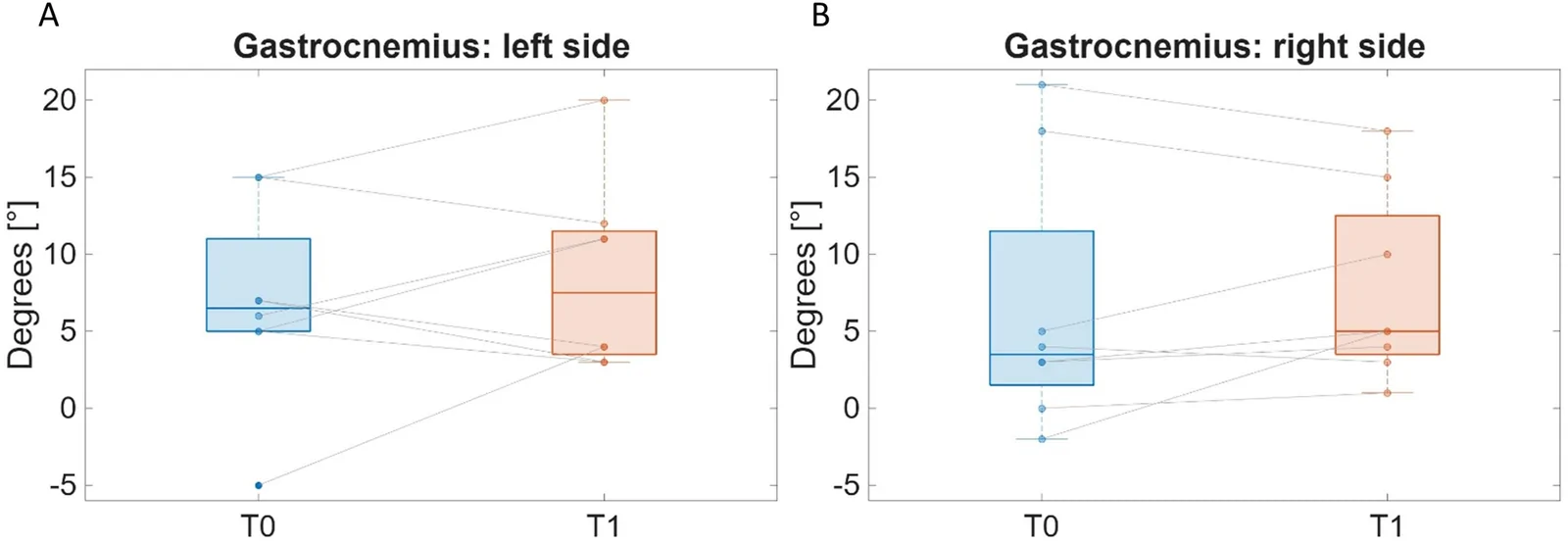
**Boxplots of muscle length measurements at baseline (T0) and post-intervention (T1) for the gastrocnemius.** Panel (A) shows results for the left lower-limb district, while panel (B) refers to the right lower-limb district. Data at T0 are displayed in blue and data at T1 in orange. Each dot represents an individual participant. Boxes indicate the interquartile range (25th – 75th percentiles), the central line represents the median, and whiskers depict the data range excluding outliers.

At group level, no statistically significant differences were observed between T0 and T1 for any muscle in either limb (all *p* > 0.05). Boxplots are shown in Figures 4–7 (panel A: left district; panel B: right district).

#### 3.4.2 Joint ROM

The ROM of knee extension and ankle dorsiflexion was assessed bilaterally at baseline (T0) and post-intervention (T1). Using a 5° threshold as a reference for clinically relevant change, individual response patterns were as follows:

- **Knee extension ROM:** left side, 1/8 (12.5%) improved, 0/8 worsened; right side, 2/8 (25%) improved, 0/8 worsened.
- **Ankle dorsiflexion ROM:** left side, 3/8 (37.5%) improved, 1/8 (12.5%) worsened; right side, 1/8 (12.5%) improved, 2/8 (25%) worsened.

Per-participant values and direction of change are reported in Table 6. Notably, participant 6, who showed the most consistent decline among functional and spasticity measures (6MWT, 10mWT, MAS), showed bilateral improvement in knee extension ROM and left-side ankle dorsiflexion, again illustrating that responses across structural, functional, and tone-related domains do not necessarily align within the same individual.

Individual ROM values for each participant are reported in Supplementary Table 1. At group level, no statistically significant differences were observed between T0 and T1 for either joint in either limb (all *p* > 0.05).

### 3.5 Balance results

Balance was evaluated through centre-of-pressure (COP) analysis on a force platform under four standing conditions: eyes open/feet apart (EOFA), eyes open/feet together (EOFT), eyes closed/feet apart (ECFA), and eyes closed/feet together (ECFT). Sway path and sway area were calculated at baseline (T0) and post-intervention (T1).

At the individual level, a reduction in sway path was observed in 6 of 8 participants (75%) in each of the four standing conditions, although the specific subset of participants showing improvement varied across conditions (participants 1, 3, 4 and 5 improved consistently across all four conditions; see Table 7 for the full pattern). Sway area changes were more variable across conditions: for EOFA, 3/8 (37.5%) improved and 5/8 (62.5%) worsened; for EOFT, improvement and worsening were equally distributed (4/8 each, 50%); for ECFA, 5/8 (62.5%) improved and 3/8 (37.5%) worsened; for ECFT, 6/8 (75%) improved and 2/8 (25%) worsened.

**Figure 8:**
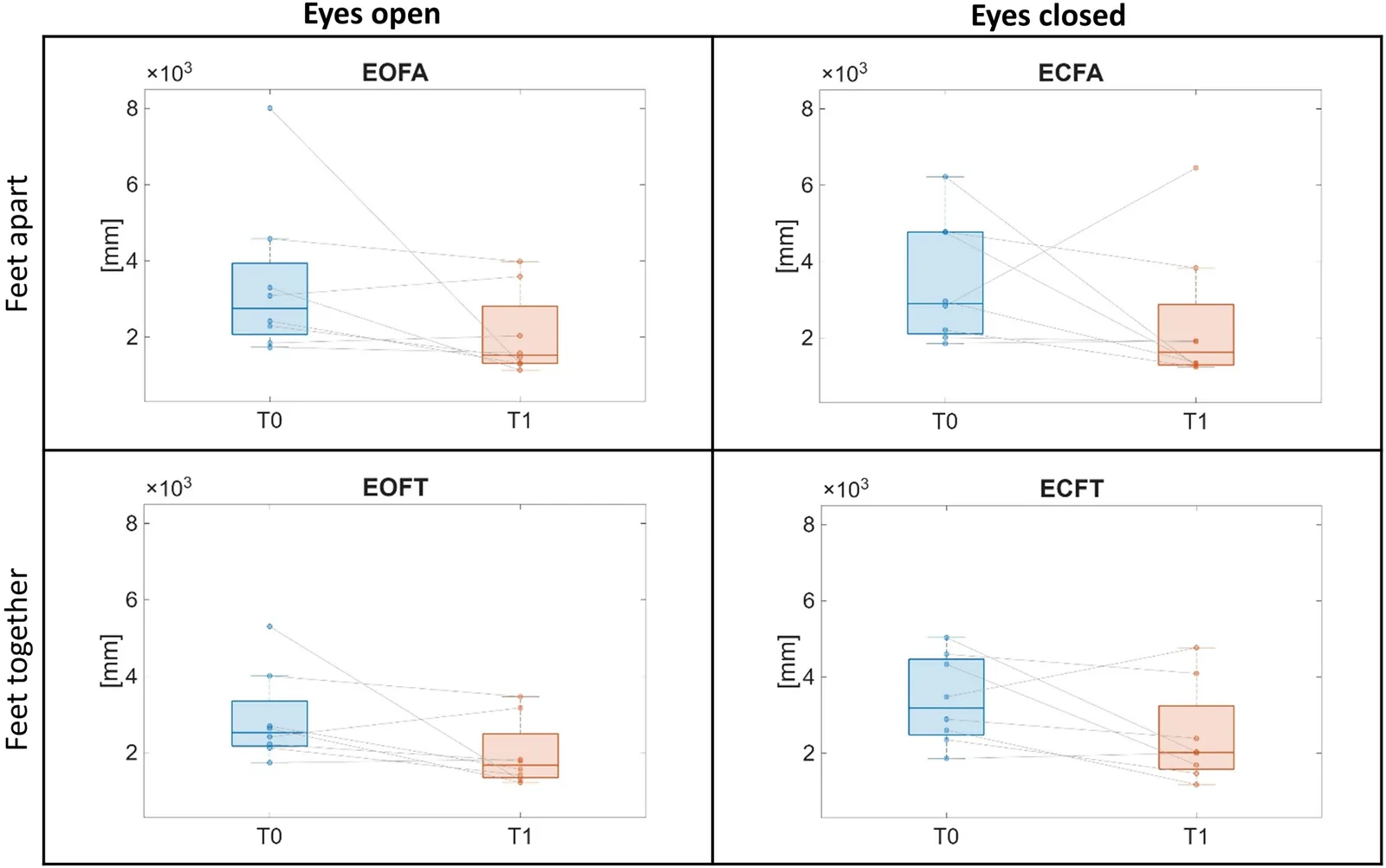
**Sway path during quiet standing under different conditions.** Boxplots of sway path measured through COP analysis on a force platform during standing with eyes open and feet apart (EOFA), eyes open and feet together (EOFT), eyes closed and feet apart (ECFA), and eyes closed and feet together (ECFT) at baseline (T0) and post-intervention (T1). Baseline data (T0) are shown in blue and post-intervention data (T1) in orange. Individual data points are displayed as dots. The box represents the interquartile range, the central line indicates the median, and whiskers indicate the data spread.

**Figure 9:**
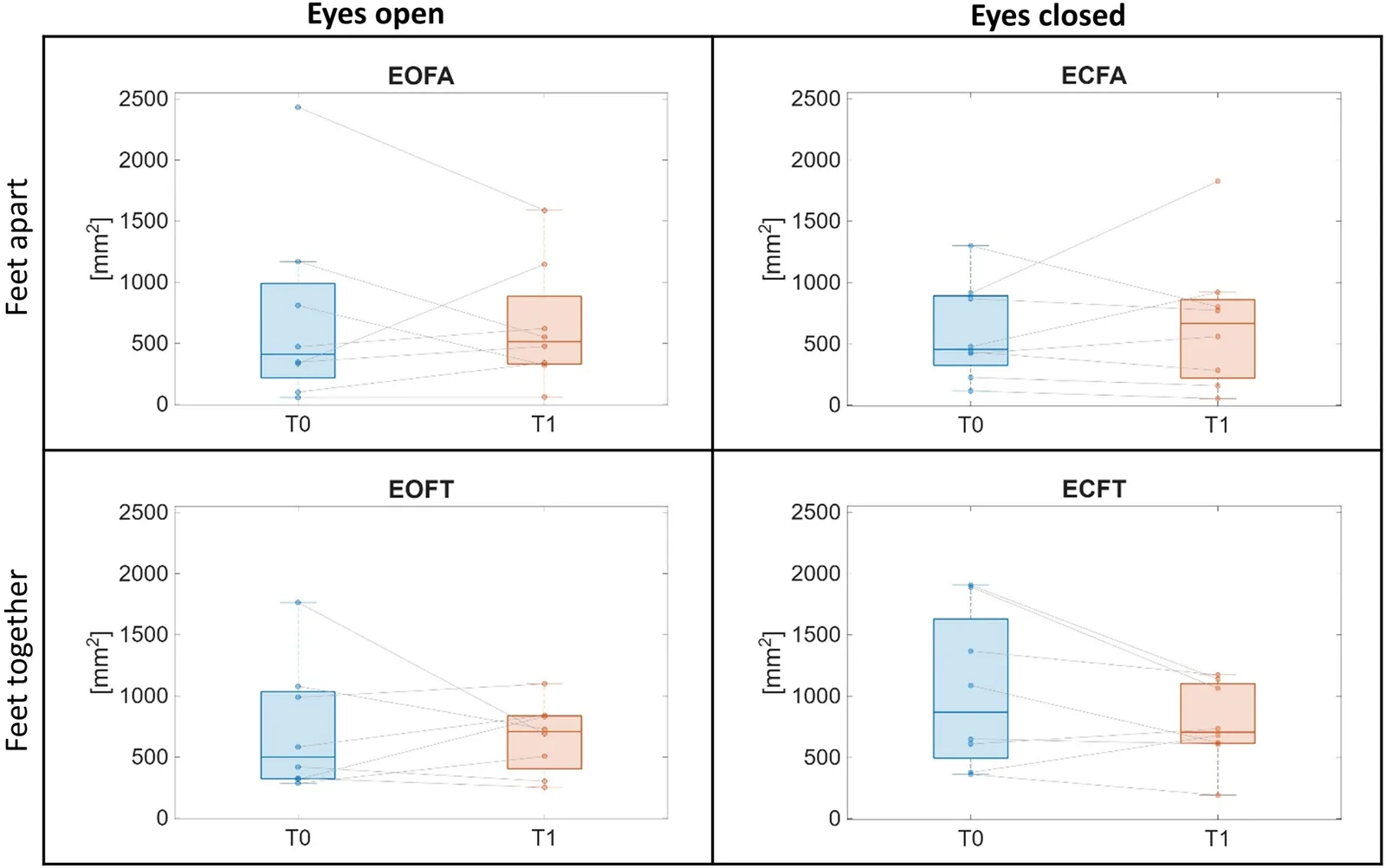
**Sway area during quiet standing under different conditions.** Boxplots of sway area measured through COP analysis on a force platform during standing with eyes open and feet apart (EOFA), eyes open and feet together (EOFT), eyes closed and feet apart (ECFA), and eyes closed and feet together (ECFT) at baseline (T0) and post-intervention (T1). Baseline data (T0) are shown in blue and post-intervention data (T1) in orange. Individual data points are displayed as dots. The box represents the interquartile range, the central line indicates the median, and whiskers indicate the data spread.

At group level, no statistically significant differences were observed between T0 and T1 for either sway path or sway area across any condition (all *p* > 0.05). Median values showed small directional variations between T0 and T1, with a tendency toward reduced sway path in some conditions, though inter-individual variability remained high (Figure 8, sway path; Figure 9, sway area).

### 3.6 Lower-limb joint ROM from 3D gait analysis through the GRAIL system

Three-dimensional gait analysis was performed using the GRAIL system to quantify sagittal-plane joint ROM of the hip, knee, and ankle during level-ground, downhill, and uphill walking. The GRAIL assessment was used both to evaluate the rehabilitative effect of Agilik (T0 vs. T1 comparisons) and to assess its assistive effect at T1 (walking without vs. with the exoskeleton).

#### 3.6.1 Evaluation of the rehabilitative effect of Agilik

Using a 5° threshold (53) as a reference for clinically relevant variation, individual-level changes were heterogeneous across joints and walking conditions:

- **Level-ground walking:** ankle ROM improved in 2/8 (25%) and worsened in 1/8 (12.5%); knee and hip ROM each improved in 1/8 (12.5%), with no clinically relevant worsening.
- **Downhill walking** (data available for 7/8 participants due to incomplete acquisition in one case): ankle ROM improved in 2/7 (28.6%) and worsened in 1/7 (14.3%); hip ROM improved in 1/7 (14.3%) and worsened in 1/7 (14.3%); no clinically relevant changes were observed at the knee.
- **Uphill walking:** ankle ROM improved in 2/8 (25%) and worsened in 1/8 (12.5%); knee ROM improved in 1/8 (12.5%) and worsened in 3/8 (37.5%); hip ROM improved in 2/8 (25%) and worsened in 1/8 (12.5%).

**Figure 10:**
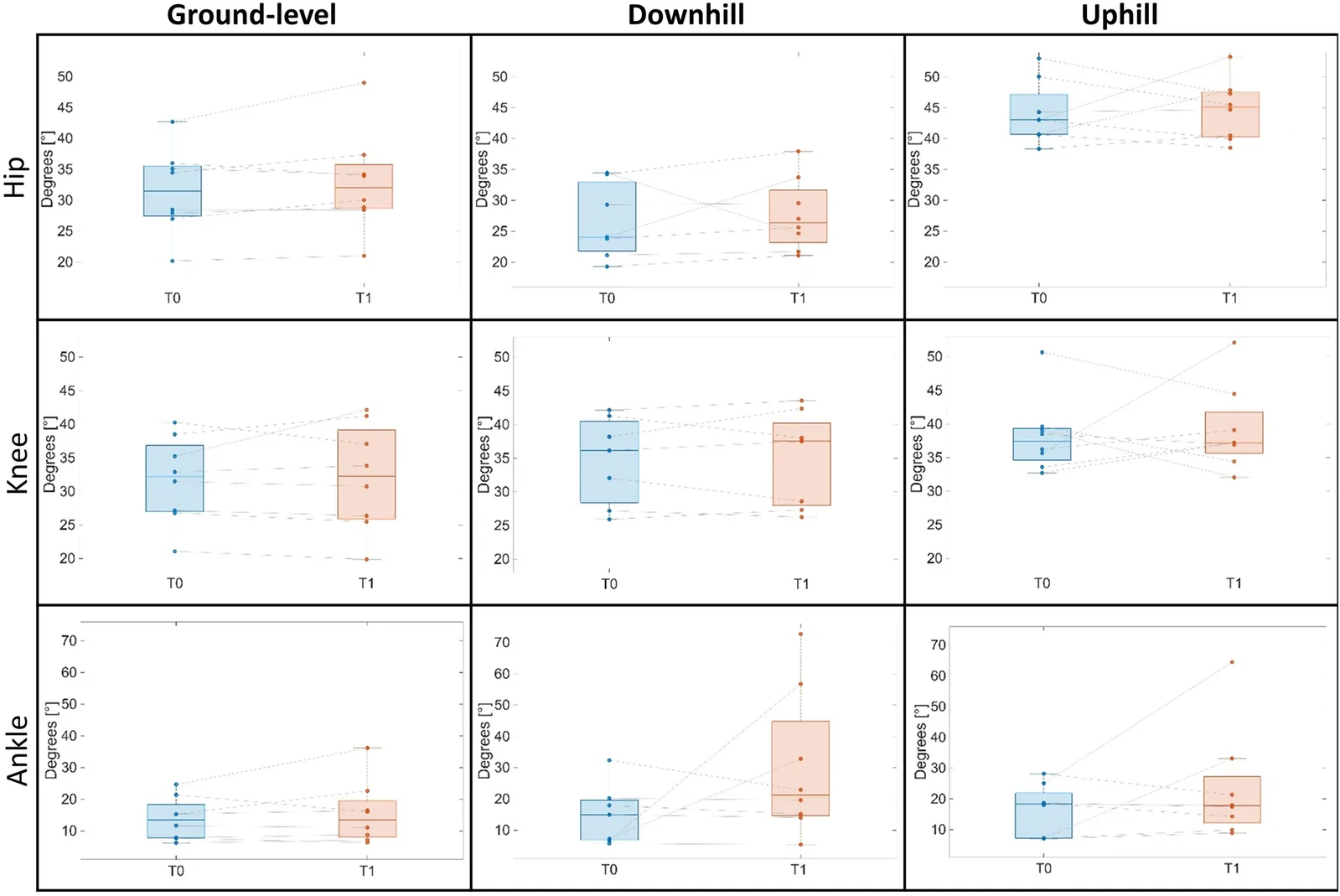
**Lower-limb joint range of motion (ROM) during gait (rehabilitative condition).** Boxplots of sagittal-plane joint ROM of the hip, knee, and ankle obtained from 3D gait analysis using the GRAIL system during level-ground, downhill, and uphill walking at baseline (T0) and post-intervention (T1). Rows correspond to the knee, hip, and ankle joints, while columns represent the different walking conditions (level-ground, downhill, and uphill). Data are averaged between the right and left lower limbs. Blue boxes represent T0 values, and orange boxes represent T1 values. Individual participant data are shown as dots. The central line indicates the median, boxes represent the interquartile range (IQR), and whiskers extend to the most extreme data points not considered outliers.

Individual-level patterns for this section are detailed in Table 8. Across all joints and walking conditions, no consistent improvement pattern was observed at the individual level, with no single joint or condition showing clinically relevant change in the majority of participants (threshold: 5°), and no statistically significant differences emerging at the group level between T0 and T1 (all *p* > 0.05); together, these findings reinforce the absence of a clear rehabilitative effect on gait kinematics during unassisted walking. Boxplots are shown in Figure 10.

#### 3.6.2 Evaluation of the assistive contribution of Agilik at post-intervention

**Figure 11:**
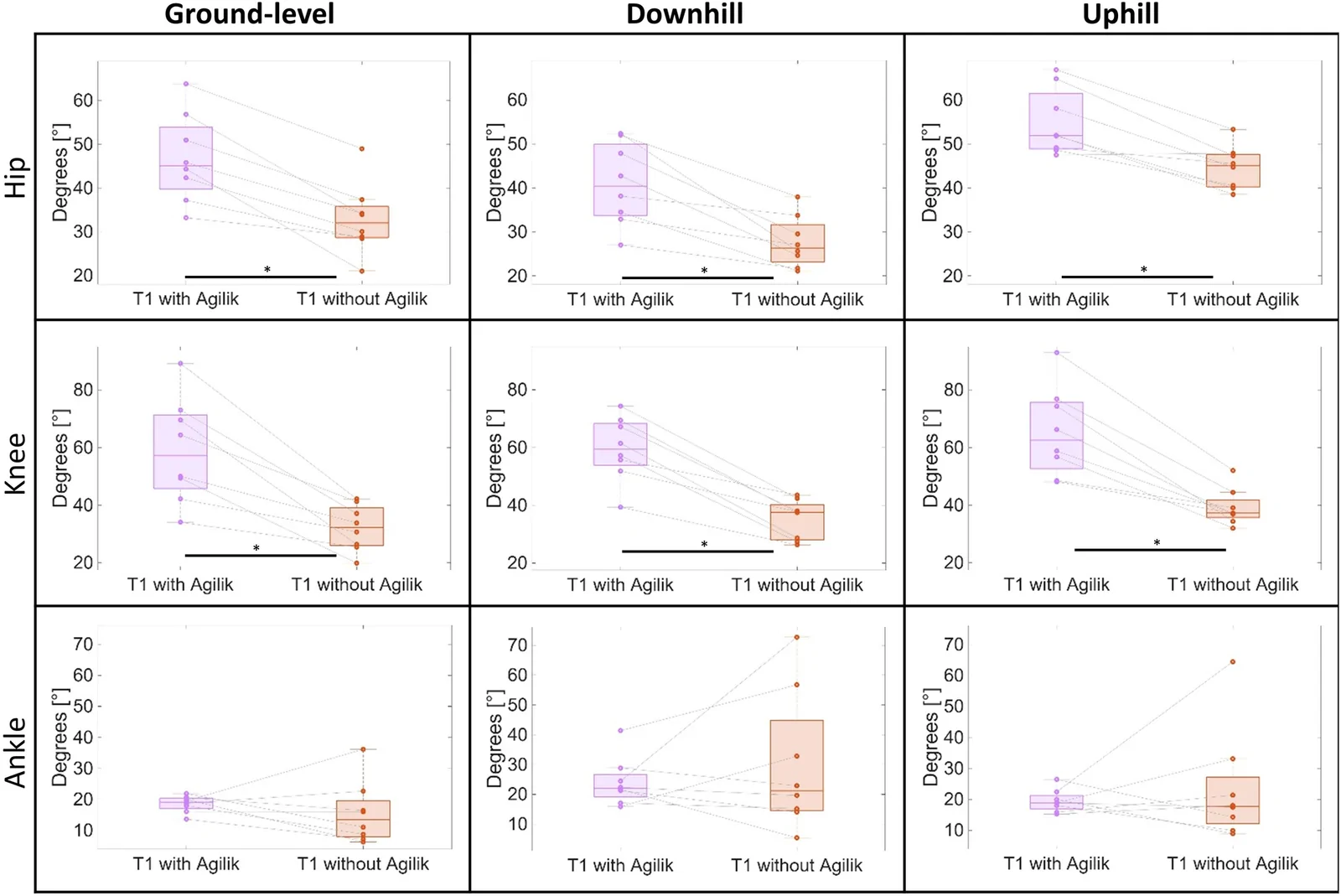
**Lower-limb joint range of motion (ROM) during gait (assistive condition).** Boxplots of sagittal-plane joint ROM of the hip, knee, and ankle obtained from 3D gait analysis using the GRAIL system during level-ground, downhill, and uphill walking at T1, without (orange) and with (violet) Agilik. Rows correspond to knee, hip, and ankle joints, while columns correspond to level-ground, downhill, and uphill walking conditions. Data are averaged between the right and left lower limbs. Violet boxes represent values collected with the exoskeleton, while orange boxes represent values collected with Agilik. Individual participant data are shown as dots. The central line indicates the median, boxes represent the interquartile range (IQR), and whiskers extend to the most extreme data points not considered outliers.

The assistive effect of Agilik on lower-limb kinematics was evaluated at T1 by comparing joint ROM during walking performed without and with the exoskeleton. Boxplots of joint ROM for the hip, knee, and ankle are reported in Figure 11. The use of Agilik resulted in a statistically significant increase in knee and hip ROM across all walking conditions (level-ground, downhill, and uphill), with all 8 participants showing improvement at the knee in every condition, and the large majority showing concordant improvement at the hip (Table 9). While the improvement in knee ROM reflects the direct actuation of the device at the knee joint, the concomitant statistically significant increase observed at the hip, despite the absence of direct actuation at this joint, suggests an indirect biomechanical effect of knee assistance on proximal lower-limb kinematics. No statistically significant differences were observed for ankle ROM between unassisted and assisted walking, consistent with the absence of direct actuation at this joint

### 3.7 User evaluation of satisfaction

**Figure 12:**
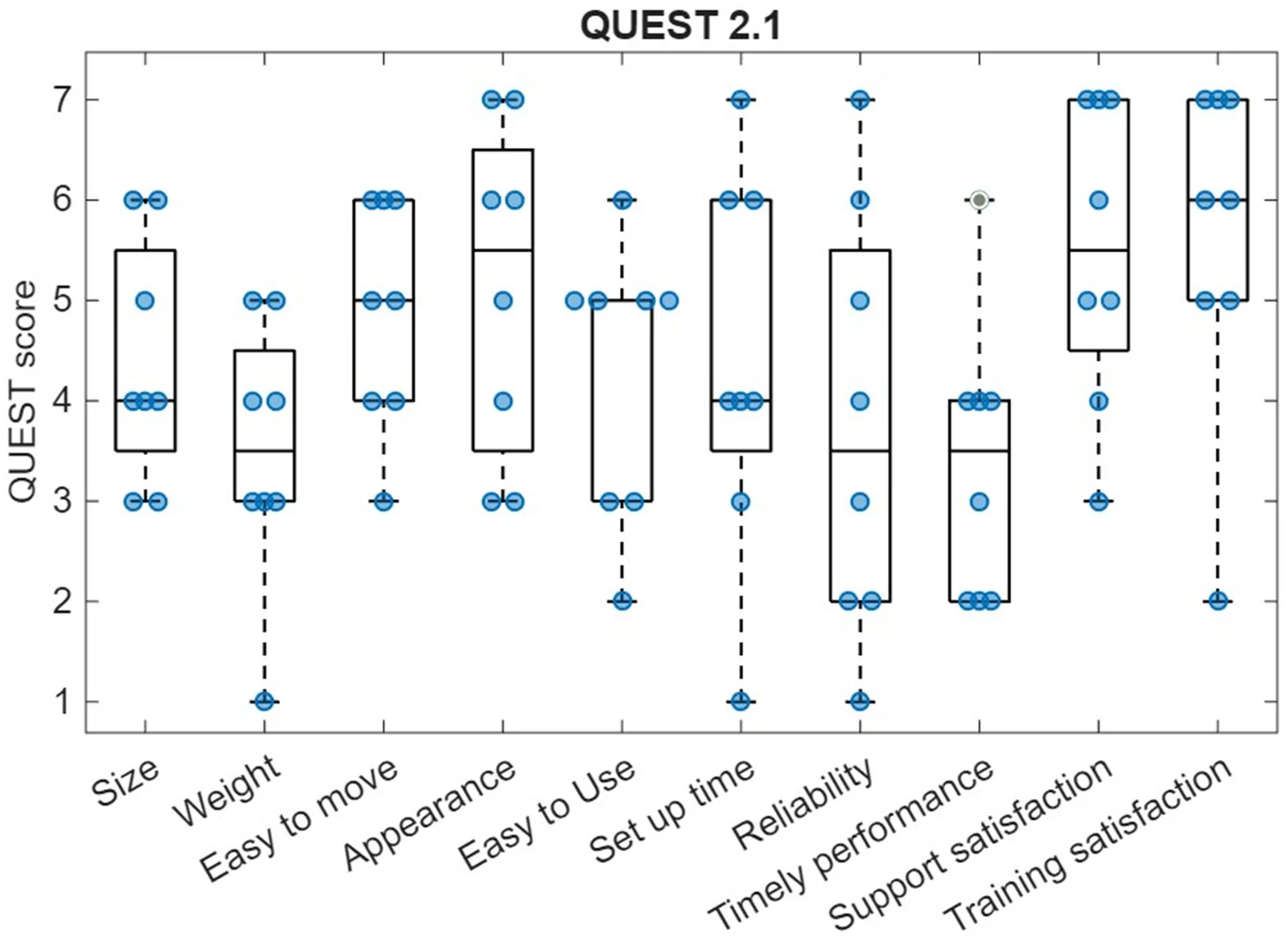
**Distribution of child-adapted QUEST 2.1 satisfaction scores at T1 for the Agilik exoskeleton.** Scores are reported on a 7-point Likert scale (1 = very dissatisfied, 7 = very satisfied). Boxes represent the median and interquartile range, and individual points show responses from the eight participants.

User satisfaction with the Agilik exoskeleton was evaluated at T1 using the child-adapted version of the Quebec User Evaluation of Satisfaction (QUEST 2.1), rated on a 7-point Likert scale. Figure 12 presents the boxplot distribution of scores for each questionnaire item, with individual data points representing the responses of the eight participants.

Overall, satisfaction ratings were moderate to high across most domains. The highest median scores were observed for Training satisfaction (median = 6) and Support satisfaction (median = 5.5), indicating a positive perception of the training programme and the support provided during the intervention. High satisfaction was reported for Appearance (median = 5.5), indicating good aesthetic acceptability of the device. With respect to usability, “Easy to move” (median = 5) and “Easy to use” (median = 5) received positive ratings, suggesting that participants found Agilik generally easy to handle during walking and training. Although the overall size of the device was judged positively (median = 4), the lower score for weight (median = 3.5) indicates that further reductions in device mass could improve user experience. Regarding device setup, the time required for configuration was perceived as acceptable (Set-up time, median = 4). In contrast, “Timely performance” (median = 3.5) and “Reliability” (median = 3.5) received lower ratings, highlighting that aspects related to device functionality and performance responsiveness represent areas for further improvement. Differences among questionnaire items were assessed using the Friedman test for related samples, which did not reveal statistically significant differences (*p* > 0.05).

When questionnaire items were aggregated into the two QUEST domains, the device-related score showed a median value of 4 (1.25), indicating moderate satisfaction with the physical and functional characteristics of the exoskeleton, whereas the service-related score was higher, with a median of 6 (1.75), reflecting a more positive evaluation of the training and support components of the intervention.

## 4 Discussion

This exploratory study investigated both the rehabilitative and assistive effects of a clinical training programme using Agilik, a wearable knee exoskeleton, in children with cerebral palsy and crouch gait. Eight participants were enrolled. This sample size is consistent with typical exploratory and pilot studies in paediatric neurorehabilitation, where small samples are commonly used to explore intervention acceptability, variability of outcome measures, and preliminary effect sizes before planning larger adequately powered studies (30, 54). Overall, the study was designed as an exploratory investigation rather than an efficacy trial and the interpretation of all outcomes below should be read accordingly: as descriptive, individual-level observations intended to inform future hypothesis-driven trials, rather than as evidence of treatment efficacy.

While no statistically significant group-level changes were observed in rehabilitative outcomes after ten training sessions, descriptive analysis at the individual level identified heterogeneous response patterns, and assistive kinematic changes were observed when the device was worn at post-intervention. Together, these findings suggest the clinical applicability of wearable knee exoskeleton training in paediatric neurorehabilitation, and provide preliminary, hypothesis-generating observations regarding individual response patterns; however, no conclusions regarding clinical effectiveness can be drawn from this study.

At the group level, no significant differences were found between baseline and post-intervention assessments for walking endurance (6MWT), walking speed (10MWT), spasticity (MAS), muscle lengths, joint range of motion, balance (COP), or lower-limb kinematics assessed through the GRAIL system. This lack of statistical significance is not unexpected given the small sample size, the heterogeneity of gait impairments typical of children with CP (55), and the limited training duration. Importantly, the absence of group-level effects does not preclude clinically meaningful changes at the individual level. Indeed, when considering minimal clinically important differences, heterogeneous response patterns were observed across participants, with a greater proportion showing improvements rather than worsening across most outcome measures. This pattern was particularly evident for functional walking capacity: three participants (participants 3, 4 and 5) exceeded the MCID for the 6MWT, and these same children also exhibited concurrent improvements in walking speed and reductions in spasticity, a co-occurrence that is reported here purely descriptively and should not be read as evidence of a treatment-related relationship, given the absence of a control group and the small sample size. Conversely, the single participant (participant 6) who showed a clinically relevant decline in 6MWT performance also demonstrated consistent worsening in 10MWT and MAS scores, suggesting internal consistency across functional measures for this individual case, again without implying a causal or treatment-related interpretation. Interestingly, this same participant showed a divergent pattern at the structural level, with improved knee extension ROM and worsened hamstring extensibility bilaterally, illustrating that a single participant's response profile can differ markedly across functional, tone-related, and structural domains.

With regard to balance outcomes, the concomitant reduction in sway path and increase in sway area observed in some participants may, speculatively, reflect a modification of postural control strategy rather than a clear deterioration in stability. Specifically, fewer corrective movements combined with a broader exploration of the base of support could indicate a more confident and less rigid postural behavior, although this interpretation remains speculative and is not supported by direct measures of postural confidence or fall risk in this study. Four participants (1, 3, 4 and 5), overlapping substantially with those showing the clearest functional gains on the 6MWT and 10mWT, showed a consistent reduction in sway path across all four standing conditions, which may be a useful signal to explore in future, larger studies examining the relationship between functional mobility gains and postural control. In this perspective, changes in COP parameters should not be interpreted solely in terms of “better” or “worse” stability, but rather as potential adaptations in motor strategy following the intervention that warrant targeted investigation in future studies.

In contrast to the rehabilitative outcomes, the assistive evaluation performed at T1 revealed clear and consistent effects of Agilik on lower-limb kinematics at the group level. When walking with the exoskeleton, participants demonstrated significantly increased knee joint ROM, reflecting the device's direct assistance on knee extensors, which are typically impaired in crouch gait. This finding is consistent with prior mechanistic and clinical work on powered knee exoskeletons in paediatric crouch gait: Lerner et al. reported improved knee extension during exoskeleton-assisted walking in children with crouch gait using a powered knee device, with related work from the same group further showing concurrent changes in lower-limb kinematics, kinetics, and muscle activity during exoskeleton-assisted knee extension (30, 54). Interestingly, a significant improvement in hip joint ROM was also observed across level-ground, downhill, and uphill walking, despite Agilik not directly actuating the hip. This finding may be compatible with inter-joint coordination effects induced by knee assistance, although the present study design does not allow causal biomechanical mechanisms to be established, and this interpretation should be regarded as hypothesis-generating only.

The absence of statistically significant changes at the ankle joint is consistent with the device design, which does not directly actuate this joint, although a small (non-significant) increase in ankle ROM was still observed across all walking conditions on average. At the individual level, however, the ankle response was markedly more heterogeneous than at the knee or hip, and was particularly non-directional during uphill walking. A single participant (participant 8) showed a consistent and comparatively large reduction in ankle ROM with the device across all three walking conditions, despite responding concordantly with the rest of the cohort at the knee and hip; this individual pattern appears to account for a meaningful share of the group-level variability at the ankle, and is reported here descriptively to illustrate the type of individual variation that aggregate statistics can obscure. This may warrant further investigation but cannot be interpreted as a reliable inter-joint effect on the basis of the present data.

These results align with previous studies reporting improvements in gait kinematics with powered knee exoskeletons and support the role of Agilik as an assistive device with the potential to enhance gait quality in real time (30, 32–34, 36, 38, 54, 56, 57). Notably, with the exception of the study by Basla et al., this is one of the few works to have evaluated the assistive effects of a knee exoskeleton not only during level walking but also during uphill and downhill gait. While Basla et al. (34) investigated these conditions using a different exoskeleton (the Myosuit) and focused on performance metrics and qualitative data, the present study extends this evidence by providing a detailed kinematic evaluation of joint ROM using 3D gait analysis on the GRAIL system. Moreover, they reported that assistance was better perceived during tasks requiring concentric muscle contractions (uphill and level walking) than during those dominated by eccentric contractions (downhill walking). In our study, Agilik was associated with measurable kinematic changes at the hip and knee across all walking conditions, including downhill gait, a pattern that may suggest that its control strategy could support joint excursion even during more demanding eccentric phases, although this remains an exploratory observation given the limited sample. However, differences in study design, device characteristics, and outcome measures limit direct comparisons.

More broadly, these assistive findings should be situated within a literature that has, to date, more consistently demonstrated immediate, device-on kinematic benefits of powered exoskeletons than durable, carry-over rehabilitative gains. Beyond the mechanistic work on knee-focused devices cited above, Choi et al. recently reported significant improvements in motor and gait outcomes following a course of overground robotic gait training with a pelvic-based device in children with cerebral palsy, in a randomized clinical trial (25). However, recent systematic reviews of robotic and exoskeleton-assisted gait training in paediatric populations have consistently highlighted that the evidence base remains constrained by small sample sizes, marked heterogeneity in participant characteristics and devices, variability in training protocols, and a generally low level of evidence across studies (17, 58). The present findings are consistent with this broader picture: a clear, statistically robust assistive effect was observed in the present study, whereas evidence for a rehabilitative, carry-over effect remains preliminary and inconclusive. This further supports our decision to frame group-level rehabilitative outcomes cautiously, and to prioritise individual-level, descriptive reporting over inferential claims regarding training efficacy.

At this stage, therefore, what the present study supports is an assistive biomechanical benefit during device use, particularly at the knee and hip, where the effect was statistically significant and consistent across all participants and walking conditions. Whether this benefit translates into a sustained rehabilitative effect after training remains an open question that cannot be addressed by the present data, and should be the primary target of future, adequately powered and controlled investigations.

In addition to the objective outcomes, usability and acceptance data provided important complementary insights. In the present study, overall satisfaction with Agilik, evaluated through the children's version of the QUEST (QUEST 2.1), was characterised by moderate acceptance of device-related features and a clearly positive perception of the training and support components. At the item level, appearance, ease of movement, and ease of use were consistently rated favourably, indicating good acceptance. These aspects are particularly relevant in paediatric rehabilitation, where comfort, aesthetics, and usability strongly influence adherence and long-term engagement. At the same time, lower scores for weight, reliability, and timely performance highlight areas requiring further technological refinement. These items relate more directly to device functionality and performance, suggesting that while the concept of wearable knee assistance is well accepted, optimisation of hardware robustness, responsiveness, and mass distribution remains essential to support long-term and potentially home-based use. These findings are broadly consistent with previous investigations of powered orthoses in paediatric populations. Devine et al. (59), who evaluated a motorized KAFO in children with crouch gait undergoing a 12-week intensive training programme, assessed satisfaction using the standard QUEST 2.0 (12 items: 8 device-related and 4 service-related, rated on a 5-point Likert scale). They reported overall high satisfaction, with service-related aspects perceived more positively than device-related characteristics. Although direct comparison is limited by differences in scale range and by our use of a child-adapted version of the questionnaire, a similar pattern emerges: across studies, the structured supervision and clinical support surrounding the intervention appear to play a central role in shaping user experience. Similarly, Hong et al. investigated satisfaction with the Angel-legs M20 (60), providing assistance at both hip and knee levels, in a heterogeneous paediatric neurorehabilitation cohort of 17 participants (12 with CP) who completed 10–23 robotic gait training sessions. Using the standard QUEST 2.0, they analysed only the eight device-related items. Most domains, including effectiveness, safety, comfort, and ease of use, were positively evaluated, whereas lower ratings were reported for weight and adjustability. This pattern closely mirrors our findings, where mass and performance-related aspects represented the main critical points. Despite differences in device architecture, target joints, and training protocols, the recurrence of weight-related concerns across studies underscores a persistent technological challenge in paediatric wearable robotics. Overall, the comparison with existing literature suggests that powered orthotic systems are generally well accepted by paediatric users and caregivers, particularly when embedded within structured and well-supported rehabilitation programmes. The practical implications of these limitations extend beyond the clinical setting: device weight and donning time directly affect a child's willingness and ability to use the exoskeleton independently or with minimal caregiver assistance in daily-life contexts, while reliability and timely performance are critical determinants of user trust and sustained engagement, particularly in paediatric populations where motivation and perceived competence strongly influence adherence. From an engineering perspective, concrete directions for improvement include the reduction of overall device mass through the adoption of lighter structural materials and more compact actuation systems, the enhancement of sensor reliability and signal processing robustness to reduce gait phase detection errors, and the development of faster and more intuitive donning procedures. In addition, the integration of adaptive control algorithms capable of automatically adjusting assistance parameters in response to real-time gait variability, rather than relying solely on manual clinician-driven parameter tuning, could improve both responsiveness and consistency of support across different walking conditions and terrains, which is particularly relevant for community and home-based use.

Despite these encouraging findings, several limitations should be acknowledged. First, the study included a small sample size (*n* = 8) and adopted a single-group pre–post design without a control group, and no formal sample size calculation or predefined primary outcome was specified *a priori*; this design was intentional and consistent with the exploratory aim of the study, but it substantially limits the ability to draw conclusions regarding the rehabilitative effectiveness of the intervention, and all findings should be regarded as hypothesis-generating rather than confirmatory. In addition, the relatively short training period may have been insufficient to induce measurable changes in several clinical outcomes, particularly considering the heterogeneity of gait impairments among children with CP. With regard to concomitant treatments and clinical profile, participant-level data were collected and are reported in Table 1. No participant had received botulinum toxin injections or undergone orthopaedic lower-limb surgery in the year preceding the study, and participants were instructed to continue their usual standard of care, including their ongoing rehabilitative and/or sport programmes and routine clinical management, so that the Agilik training represented the only planned modification to participants' treatment. However, the data reveal meaningful heterogeneity in background physiotherapy intensity (ranging from no ongoing physiotherapy to 6–7 sessions per week) and in orthotic management, which varied from no orthoses to bilateral AFOs with walking aids. These inter-individual differences in background treatment and clinical profile represent a relevant source of variability in treatment response that cannot be fully controlled in a single-group exploratory study, and should be prospectively and systematically documented in future studies. Furthermore, exoskeleton parameter adjustments were based on clinical observation and expert judgement rather than a standardised optimisation protocol. Although this individualised approach reflects current clinical practice and allowed assistance to be tailored to each participant's gait pattern and therapeutic needs, it limits the reproducibility of the intervention across clinicians and centres. Future studies should therefore develop and validate standardised parameter-setting procedures, particularly for paediatric populations, to improve intervention consistency and facilitate comparison across studies. Finally, the absence of long-term follow-up does not allow conclusions regarding the persistence of either the rehabilitative or assistive effects observed. Future randomized controlled studies involving larger cohorts, longer intervention periods, systematic documentation of concomitant treatments and orthopaedic history, and longitudinal follow-up are therefore warranted to confirm these preliminary findings and better define the clinical role of wearable knee exoskeletons in paediatric neurorehabilitation.

## 5 Conclusions

Taken together, these findings indicate that Agilik is applicable and well tolerated in a paediatric clinical setting, provides measurable assistive benefits on lower-limb kinematics, and is well accepted by users. These conclusions are necessarily preliminary and descriptive, consistent with the exploratory design and small sample size of this study. The absence of statistically significant rehabilitative effects after ten sessions should be interpreted accordingly, rather than as evidence of inefficacy. Short training duration, limited statistical power, and inter-individual variability are likely contributors to the observed results, as further illustrated by the heterogeneous individual response patterns described above.

This study provides important methodological insights for the design of future trials, including the selection of sensitive outcome measures, the importance of MCID-based and individual-level analyses in small, heterogeneous cohorts, and the need for longer interventions and larger samples. In line with this, future studies should prospectively and systematically document participants' orthopaedic profile, orthotic management, and concomitant treatments (including physiotherapy intensity and pharmacological interventions) as these variables showed meaningful inter-individual variability in the present cohort and may represent relevant sources of heterogeneity in treatment response. Future studies should include controlled designs, extended training periods, and follow-up assessments to determine whether repeated exposure to assisted gait can translate into sustained functional improvements.

In conclusion, what this exploratory study has demonstrated is an assistive biomechanical benefit of Agilik during device use, particularly at the knee and hip, which was statistically significant, consistent across participants, and robust across multiple walking conditions. Individual-level, descriptive observations further suggest that some participants may show clinically meaningful changes across functional and structural outcomes following training; however, these remain hypothesis-generating findings that cannot be interpreted as evidence of a rehabilitative effect without confirmation in larger, controlled studies. This study provides a foundation for future larger-scale, controlled investigations aimed at establishing the long-term clinical effectiveness of wearable knee exoskeletons in children with cerebral palsy and crouch gait.

## Data Availability

The datasets presented in this study can be found in online repositories. The names of the repository/repositories and accession number(s) can be found below: Link to the Zenodo repository: https://zenodo.org/records/21100298.
