# Supplementary material for: Evaluating the rehabilitative and assistive impact of the Agilik exoskeleton: a pre–post single-group study in children with cerebral palsy
Source: Front Med (Lausanne). 2026 Sep 9;13:1814298. doi: 10.3389/fmed.2026.1814298 (PMC13597934; doi:10.3389/fmed.2026.1814298)
Supplement: Supplementary file 1 [file Table1.docx]

**Supplementary Table 1.** Individual sagittal-plane knee extension and ankle dorsiflexion range of motion (°), assessed bilaterally (left and right limbs) at baseline (T0) and post-intervention (T1).

| **Subject** | **Time point** | **Knee extension [°]** | | **Ankle dorsiflexion [°]** | |
| --- | --- | --- | --- | --- | --- |
|  |  | **Left side** | **Right side** | **Left side** | **Right side** |
| 1 | T0 | 0 | 2 | 20 | 34 |
| 1 | T1 | 0 | 0 | 27 | 25 |
| 2 | T0 | 0 | 2 | 5 | 2 |
| 2 | T1 | 0 | 1 | 19 | 13 |
| 3 | T0 | -4 | -5 | 15 | 5 |
| 3 | T1 | -5 | 2 | 14 | 8 |
| 4 | T0 | 4 | 8 | 16 | 21 |
| 4 | T1 | 2 | 4 | 14 | 15 |
| 5 | T0 | -8 | -8 | 20 | 18 |
| 5 | T1 | -6 | -9 | 15 | 15 |
| 6 | T0 | -7 | -8 | 6 | 13 |
| 6 | T1 | 0 | 0 | 12 | 12 |
| 7 | T0 | 3 | 0 | 22 | 17 |
| 7 | T1 | 1 | 0 | 22 | 16 |
| 8 | T0 | -2 | -1 | 28 | 28 |
| 8 | T1 | -3 | -3 | 30 | 30 |
